# Acute Interventions for Posttraumatic Stress Disorder and Alcohol Misuse in Women Exposed to Sexual Assault: Protocol for a Phased Randomization Trial (Project SARAH)

**DOI:** 10.2196/107568

**Published:** 2026-11-03

**Authors:** Michele A Bedard-Gilligan, Elizabeth A Lehinger, Molly Joseph, Debra L Kaysen, Cynthia A Stappenbeck, Jennifer M Cadigan, Charles B Fleming, Lori A Zoellner

**Affiliations:** 1 Department of Psychiatry and Behavioral Sciences, School of Medicine, University of Washington, 1959 NE Pacific St, Box 356560, Seattle, WA, 98195, United States, 1 2066164215; 2 Department of Psychiatry, School of Medicine, Stanford University, Palo Alto, CA, United States; 3 Department of Psychology, Georgia State University, Atlanta, GA, United States; 4 Department of Psychology, University of Washington, Seattle, WA, United States

**Keywords:** sexual assault, PTSD, alcohol misuse, randomized trial, fear, reward

## Abstract

**Background:**

Sexual assault can have devastating consequences for survivors, including posttraumatic stress disorder (PTSD) and alcohol misuse. Interventions provided in the initial months following a sexual assault can prevent the development of chronic problems. Early interventions that are brief, accessible, and focused on shared vulnerabilities may improve efficacy.

**Objective:**

Project SARAH (Sexual Assault Recovery and Hope) is designed to test fear and reward learning as processes underlying recovery following sexual assault and to elucidate efficacious approaches for brief interventions to address PTSD and alcohol misuse.

**Methods:**

A phased, doubly randomized, and single-masked clinical trial is being conducted to test the efficacy of acute intervention approaches that target (1) the fear system underlying PTSD with imaginal exposure or (2) the reward system underlying alcohol use with alcohol skills training, comparing these approaches to supportive counseling via telehealth for reducing PTSD and problematic drinking. The interventions are tested in a sample of adult female sexual assault survivors. The phased randomization study design allows for comparisons between imaginal exposure, alcohol skills training, and their combination with the active control, supportive counseling, as well as to test mechanisms of recovery.

**Results:**

The study was funded in 2019. After modifications to include remote procedures, necessitated by the COVID-19 pandemic, participant enrollment began in March 2021 and ended in December 2025, and our final follow-up was completed in May 2026. We enrolled 82 participants. Analysis of main outcomes (PTSD severity and alcohol use) and dissemination of main outcomes are expected by the summer of 2027.

**Conclusions:**

This design is significant for exploring PTSD and alcohol recovery pathways acutely following sexual assault.

## Introduction

Many survivors of sexual violence develop posttraumatic stress disorder (PTSD) and alcohol misuse [1]. In the months following assault, symptoms decrease naturally, but a significant minority of survivors (41%) meet criteria for PTSD 1 year postassault [2]. Heavy alcohol use is associated with a lower probability of recovery [3], and PTSD predicts the development of alcohol use disorders (AUDs) [4,5], especially for women [6]. PTSD plus AUD results in increased health care use, greater impairment, and higher treatment dropout [7,8]. Thus, we need accessible treatments for sexual assault survivors with PTSD and alcohol misuse.

The acute phase of recovery, weeks to a year postassault, is critical. Before symptoms become entrenched, we can reduce the dose of intervention and potentially increase retention and engagement in interventions [9,10]. Cognitive behavioral therapy–based interventions delivered acutely after traumatic events are effective in reducing PTSD [11], although there is currently no gold-standard approach [12]. Importantly, improvements in chronic PTSD predict improvements in heavy drinking and vice versa [13,14], but acute interventions have not yet targeted the co-occurrence of PTSD and alcohol misuse.

Theories on the co-occurrence of PTSD and alcohol misuse posit shared mechanisms [15,16] and functional relationships between distress and alcohol use (eg, self-medication [17]). Impaired fear learning, including impaired fear inhibition [18-20] and extinction [21], is consistently observed in people with PTSD, and PTSD treatments aim to promote extinction learning, which is a primary mechanism through which treatments are theorized to be effective [22,23]. Relatedly, alcohol use affects contextual fear conditioning [24] and also impairs fear extinction learning [25]. Similarly, impaired reward learning is implicated in PTSD and alcohol misuse. Both are characterized by dysfunction in pursuing, receiving, and experiencing pleasure [26-28]. Alcohol interventions commonly include strategies to increase healthy reward-seeking [29,30]. Although fear and reward systems work in tandem to avoid threats and obtain rewards [27,31], little human research has examined the integration of fear and reward systems [32] despite their possible relevance to the co-occurrence of PTSD and AUD.

Studies conducted acutely post trauma, while symptoms and functional relationships are being established, can better isolate specific vulnerabilities. We designed the current study to answer critical questions related to efficacious treatment delivery and outcomes by using a phased randomized intervention design to compare interventions targeting fear and reward impairment among female-identifying sexual assault survivors. The inclusion of objective behavioral fear and reward tasks, in addition to self-report measures, will add to our understanding of treatment mechanisms and their role in reducing PTSD and alcohol misuse. This study uses a novel phased design to answer the following clinically relevant questions:

1. Do early interventions need to target both PTSD and alcohol use, or just one?
2. Does the order in which PTSD and alcohol interventions are delivered matter?
3. For whom are one or both treatments needed?

The specific research aims of this study are to (1) evaluate whether targeting PTSD via imaginal exposure (IE) or alcohol misuse via alcohol skills training (AST) is most effective compared to supportive counseling (SC); (2) evaluate whether targeting PTSD or alcohol misuse alone (IE or AST) or in combination is most efficient; and (3) evaluate individualized prediction of intervention response, examining baseline performance on fear and reward tasks and time since assault, as differential intervention moderators (IE vs AST). Time since assault is included given the known effects of time on natural recovery of symptoms (ie, symptoms tend to decrease naturally over time). Due to the COVID-19 pandemic, shifts to the originally proposed procedures to change to remote delivery of assessments, behavioral tasks, and interventions were needed. This paper describes the original protocol for this study, including design, measures, procedures, and analysis plan, as well as pandemic-necessitated shifts.

The guiding hypotheses are as follows:

- H1 (aim 1): patients assigned to IE or AST will show greater decreases in PTSD and alcohol use than individuals in SC.
- H2 (aim 1): IE will produce larger decreases in PTSD symptoms, and AST will produce greater decreases in alcohol use.
- H3 (aim 2): participants assigned to receive both IE and AST will show greater decreases in PTSD and alcohol use than individuals assigned to only one of these interventions.
- H4 (aim 3): baseline impairments in fear processing will predict greater PTSD, and baseline reward deficits will predict greater alcohol use.
- H5 (aim 3): improvements in PTSD associated with IE will be larger for people with greater baseline impairments in fear processing, and reductions in alcohol use associated with AST will be larger for people with baseline impairments in reward.
- H6 (aim 3): less time since the assault will be associated with a larger response to receiving only 1 intervention (IE or AST).

## Methods

### Trial Design

Project SARAH (Sexual Assault, Recovery and Hope; NCT04124380) is a phased, doubly randomized, single-masked clinical trial exploring the comparative efficacy of 3 brief interventions delivered in the acute phase following sexual assault: IE (6 sessions), AST (6 sessions), and SC (12 sessions). IE and AST participants are doubly randomized to examine combined vs individual treatments and the order of treatment delivery (exploratory). Furthermore, we examine fear and reward processes via behavioral tasks and self-report measures. Due to the stay-at-home orders and cautionary guidelines related to the COVID-19 pandemic, the original in-person procedures were shifted to accommodate video and telephone delivery. The protocol follows the SPIRIT (Standard Protocol Items: Recommendations for Interventional Trials) 2025 guidelines for reporting in clinical trials (Checklist 1).

### Participants

#### Overview

The original design aimed to enroll 180 female-identifying individuals, aged 18 to 65 years, who reported experiencing a sexual assault between 4 weeks and 1 year prior to enrollment, and were above thresholds for PTSD and alcohol misuse (see Textbox 1 for inclusion/exclusion criteria). Sexual assault was defined as any experience of unwanted sexual contact, such as attempted or completed sexual touching or rape (eg, oral, anal, or vaginal penetration [1]). The original start date for enrollment was April 2020, but the COVID-19 pandemic delayed recruitment until March 2021.

Textbox 1. **Inclusion/exclusion criteria.**

**Inclusion criteria**

- Identifies as female
- Between the ages of 18 and 65 years
- Reports a sexual assault between 4 weeks and 1 year prior to enrollment
- Current posttraumatic stress disorder severity of 23+ on the PTSD Symptom Scale–Interview for DSM-5
- Current heavy alcohol use (2+ heavy episodic drinking [4+ drinks] occasions in the past month)

**Exclusion criteria**

- Current diagnosis of schizophrenia, delusional disorder, or organic mental disorder as defined by the *DSM-5* (*Diagnostic and Statistical Manual of Mental Disorders* [Fifth Edition])
- Current diagnosis of bipolar disorder, depression with psychotic features, or depression severe enough to require immediate psychiatric treatment (ie, serious suicide risk with intent and plan)
- Unwilling or unable to discontinue current trauma-focused psychotherapy or current substance use psychotherapy
- Unstable dose of psychotropic medications in the prior 3 months
- Ongoing intimate relationship with the perpetrator of most recent assault
- Current diagnosis of a severe substance use disorder according to *DSM-5*, other than alcohol in the last month
- No clear trauma memory
- Current higher dose use of benzodiazepines (greater than the equivalent of 4 mg of lorazepam, 2 mg alprazolam, 1.5 mg clonazepam, or 20 mg of diazepam)

#### Recruitment

Participants were recruited using (1) referrals from clinics, community providers, and emergency rooms; (2) advertisements in newspapers, online classifieds, and on social media; (3) flyers and brochures in local venues (eg, coffee shops and community centers); and (4) emails to enrolled university students. Recruitment originally focused on Western Washington. When the study launched online, recruitment extended across Washington State. Due to slower-than-anticipated recruitment, we began recruitment in California in February 2024 using the same strategies. Recruitment remained slow, however, resulting in a smaller sample than intended.

### Measures

Additional outcome measures are listed in Table 1, including a description of outcome type, timing, and administration method.

**Table 1.** Overview of assessment measures and their administration^a^.

Purpose	Measure	Time frame, administration, and time points		
Primary outcomes				
PTSD^b^ severity	PSS-I-5^c^ [33] PSS-SR-5^d^ [34]	PSS-I-5 time frame: past 2 weeks; administration: interview; time point: baseline, follow-ups PSS-SR-5 time frame: past 2 weeks; administration: self-report; time point: baseline, follow-ups PSS-SR-5 time frame: past week; administration: self-report; time point: weekly		
Alcohol use: frequency and quantity, HED^e^	TLFB^f^ [35]	Time frame: past month; administration: interview; time point: baseline, follow-ups		
Alcohol use: typical frequency and quantity	DDQ^g^ [36]	Time frame: past month; administration: self-report; time point: baseline, follow-ups Time frame: past week; administration: self-report; time point: weekly		
Secondary outcomes				
Depression	QIDS-C^h^ [37] QIDS-SR^i^ [37]	QIDS-C time frame: past 2 weeks; administration: interview; time point: baseline, follow-ups QIDS-SR time frame: past week; administration: self-report; time point: baseline, follow-ups, weekly		
Alcohol consequences	SIP^j^ [38]	Time frame: lifetime; administration: self-report; time point: baseline, follow-ups		
Fear/avoidance	PABQ^k^ [39]	Time frame: since trauma; administration: self-report; time point: baseline, follow-ups		
Alcohol cravings	PACS^l^ [40]	Time frame: past week; administration: self-report; time point: baseline, follow-ups, weekly		
Reward/anhedonia	SHAPS^m^ [41]	Time frame: past few days; administration: self-report; time point: baseline, follow-ups, weekly		
Psychosocial functioning	GPF^n^ [42]	Time frame: past week; administration: self-report; time point: baseline, follow-ups, weekly		
Quality of life functioning	GQL^o^ [42]	Time frame: current; administration: self-report; time point: baseline, follow-ups, weekly		
Additional measures (inclusion, mechanisms, and symptoms)				
Fear learning	AX+/BX– [43]	Time frame: N/A; administration: experimental task; time point: baseline		
Reward learning	PRT^p^ [44]	Time frame: N/A; administration: experimental task; time point: baseline		
Suicidality	C-SSRS^q^ [45]	Time frame: lifetime; administration: interview; time point: baseline Time frame: past month; administration: interview; time point: baseline, follow-ups		
Other mental disorders	SCID-5^r^ [46]	Time frame: lifetime; administration: interview; time point: baseline Time frame: current; administration: interview; time point: baseline, 6-month follow-up		
Unwanted sexual experiences	SES^s^ [47]	Time frame: lifetime, past 12 months; administration: self-report; time point: baseline Time frame: since enrollment; administration: self-report; time point: 6-month follow-up		
Drinking motives	DMQ^t^ [48]	Time frame: lifetime; administration: self-report; time point: baseline, follow-ups		

a Interview measures are administered on Zoom, with interviewers entering responses into measure documents for later data entry by the study coordinator; self-report measures are completed by participants directly in a survey link while on a Zoom session monitored by the study coordinator for any questions and for compliance by the participant.

b PTSD: posttraumatic stress disorder.

c PSS-I-5: PTSD Symptom Scale–Interview for DSM-5.

d PSS-SR-5: PTSD Symptom Scale–Self-Report.

e HED: heavy episodic drinking.

f TLFB: Timeline Followback.

g DDQ: Daily Drinking Questionnaire.

h QIDS-C: Quick Inventory of Depressive Symptomatology–Clinical Rating.

i QIDS-SR: Quick Inventory of Depressive Symptomatology–Self-Report.

j SIP: Short Inventory of Problems.

k PABQ: Posttrauma Avoidance Behavior Questionnaire.

l PACS: Penn Alcohol Craving Scale.

m SHAPS: Snaith-Hamilton Pleasure Scale.

n GPF: Global Psychosocial Functioning.

o GQL: Global Quality of Life.

p PRT: Probabilistic Reward Task.

q C-SSRS: Columbia Suicide Severity Rating Scale.

r SCID-5: Structured Clinical Interview for DSM-5.

s SES: Sexual Experiences Survey.

t DMQ: Drinking Motives Questionnaire.

#### Demographics and Background Characteristics

Demographics (eg, age, sexual orientation, race, ethnicity, education, and treatment history) are assessed via interview. The Trauma History Questionnaire [49] assesses lifetime trauma exposure. Both treatment and trauma exposure are reassessed at the 6-month follow-up.

#### Interview Measures: Primary Outcomes

The primary outcome measures are as follows:

- PTSD Symptom Scale–Interview for DSM-5 (PSS-I-5) [33]: this 20-item interview rates *DSM-5* (*Diagnostic and Statistical Manual of Mental Disorders* [Fifth Edition]) symptoms on a 0 to 4 scale of frequency/intensity for the last 2 weeks. Symptoms are anchored to the recent sexual assault. Scale scores are the sum of all item scores. The PTSD Symptom Scale–Interview (PSS-I) shows good interrater reliability, comparable psychometrics to other interviews, and a shorter administration time [33]. The scale score will be used for analyses of condition differences (primary outcome).
- Timeline Followback (TLFB) [35]: the TLFB, a calendar-based assessment of drinking, is used to assess quantity and frequency of alcohol use over the prior 30 days, including the number of drinking days and number of drinks consumed on drinking days (primary outcomes). Additional measures of alcohol use behavior include the peak number of drinks and the number of heavy episodic drinking (HED) days. The TLFB interview shows high reliability and validity [50].

#### Weekly Self-Report Primary Outcome Measures

During the 2 active intervention phases (phase 1: 3 weeks; phase 2: 3 weeks), participants are administered weekly measures of PTSD and alcohol use. Participants complete the measures online. Weekly self-reports of PTSD symptoms and drinking are included as primary outcomes as follows:

- PTSD Symptom Scale–Self-Report (PSS-SR-5) [33]: the PSS-SR-5 is a 20-item measure that queries for a self-report assessment of the frequency/intensity of the 20 *DSM-5* PTSD symptoms. Participants rate symptoms they experienced in the past week. As with the PSS-I-5, scale-scores are the sum of the 20 item scores.
- Daily Drinking Questionnaire (DDQ) [36]: the weekly version of the DDQ asks participants to report on their alcohol use in the past week. To capture both frequency and quantity of alcohol consumption, we will calculate measures of the number of days of drinking in a typical week and the total number of drinks consumed in a typical week.

#### Behavioral Tasks

The behavioral tasks used in this study were adapted for online administration.

##### Baseline Fear Learning: Discriminative Conditioning Task

The Discriminative Conditioning Task (AX+/BX–) [43] is well established and assesses fear conditioning and extinction by capturing participants’ expectancy ratings for conditioned stimuli. In this paradigm, conditioned stimuli are presented to participants as counterbalanced colored shapes (eg, blue triangle and yellow square), represented by the terms A, B, and X. An aversive unconditioned stimulus (US), a standardized audio of a scream and the image of a screaming lady chosen for remote application and due to its common usage in PTSD studies [51], is paired with X in the presence of A but not B. Thus, the A stimulus predicts the fear response as it is associated with the US (danger), while the B stimulus is inhibitory as it is associated with the absence of the US (safety). The danger/safety associations (ie, AX and BX) are learned through 36 training trials (6s each). After conditioning, participants complete a critical transfer trial where they are given the ambiguous combination of a safety signal (B) and a danger signal (A). Participants then complete extinction trials (72 trials) in which no stimuli are paired with the US. In this online version, fear response is operationalized as self-reported expectancy and distress (1-7; very unlikely/not at all distressing to very likely/extremely distressing) during the conditioning, AB transfer trials, and extinction trials. The inclusion of psychophysiological indicators was not feasible due to online administration.

##### Baseline Reward Learning: Probabilistic Reward Task

The Probabilistic Reward Task (PRT) measures reward learning and functioning based on learned preference for one rewarded stimulus over another less rewarded stimulus. It is a well-validated assessment of reward learning [44] and a marker of reward functioning [52]. The stimuli are 2 simple line faces (diameter: 25 mm; eyes: 7 mm) differentially associated with monetary reward versus no reward. At the start of each trial, the face has no mouth. After a delay, either a “short mouth” measuring 11.5 mm or a “long mouth” measuring 13 mm is presented for 100 ms. Participants must decide whether a long or small mouth was presented. Across participants, short and long mouths are counterbalanced as either “rich” or “lean” conditions, where correct responding to the rich stimuli is rewarded at a rate of 3 times more than the lean stimuli (*“*Correct! You won 20 cents”). There are three blocks with 100 trials each. Response bias is the tendency to select one stimulus over the other, with a preference for the stimulus more often rewarded. Reward propensity is calculated by increases in response bias during the final block relative to the first block.

### Procedures

Figure 1 shows the flow of the study procedures, including intake, intervention delivery, and assessments.

**Figure 1.**
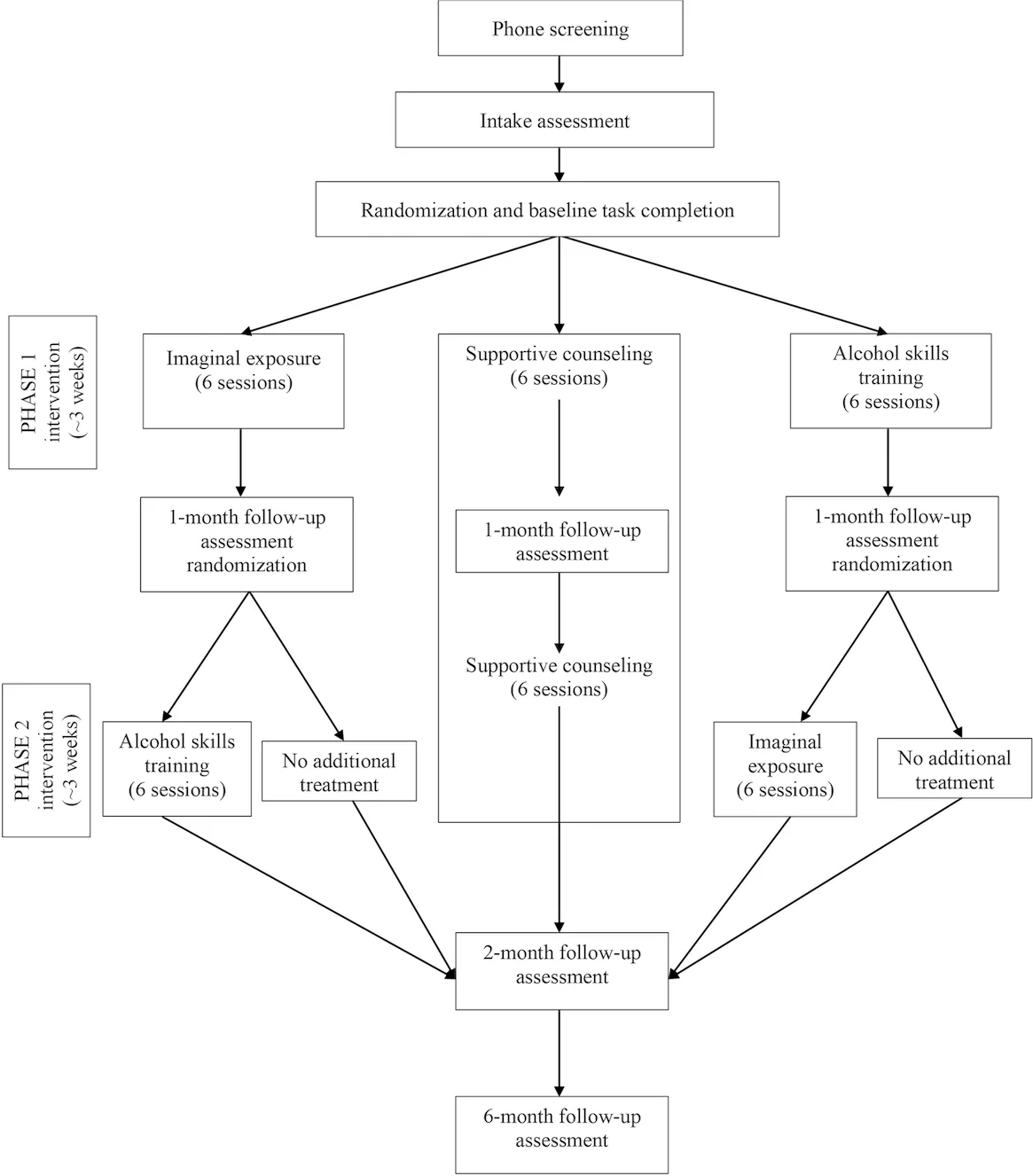
Overview of study procedures.

#### Screening and Intake

Participants are screened on the phone for potential eligibility. Individuals reporting sexual assault, PTSD symptoms, and alcohol use are scheduled for a 2 to 3-hour intake with an assessor over Zoom. Informed consent is obtained and semistructured interviews are administered. Intake assessments are presented to the clinical team (ie, assessors and clinicians) to determine final eligibility.

#### Randomization, Experimental Tasks, and Baseline Self-Reports

At randomization appointments, behavioral tasks are administered and self-report surveys are completed via Qualtrics. Participants remain on video for the duration of the appointment with the study coordinator, who is available for questions and monitors engagement. Next, the coordinator uses a custom-built randomization program to assign participants to their initial intervention condition (1:1:1 for IE, AST, or SC). Two stratification variables are used: PTSD severity (PSS-I-5 total ≥36 vs<36) and alcohol use severity (≥6 vs<6 drinking days in the past 2 weeks) to ensure representation of severity across conditions. The randomization algorithm adjusts probabilities based on prior assignments to ensure a similar number of participants in each condition. Assessors stay masked to condition assignments throughout the study.

#### Intervention Delivery and Rerandomization

Participants assigned to IE or AST complete 6 sessions across 3 weeks (phase 1), followed by rerandomization at 1-month postintake to the other active intervention or no additional intervention (phase 2). The second randomization occurs for all participants in the IE and AST conditions, regardless of whether they complete phase 1 intervention sessions or the 1-month postintake assessment. For the second randomization, participants have a 0.5 probability of assignment to the other intervention. Those assigned to the other intervention receive 6 sessions across 3 weeks with a new therapist. Participants assigned to SC complete 6 sessions across 3 weeks (phase 1) and then an additional 6 sessions across 3 weeks (phase 2) with the same therapist. Across both phases, participants complete weekly online self-report surveys assessing alcohol use, PTSD, depression, and fear and reward processes (ie, before intervention sessions 1, 3, and 5; once per week for those assigned to no intervention in phase 2). These weekly assessments are used to model symptom change and monitor treatment progress.

#### Follow-Up Assessments: Interviews and Self-Reports

At 1 month, 2 months, and 6 months following randomization, participants meet with the same assessor from intake and complete interviews and online self-report questionnaires. Again, a study coordinator is present during the appointments. At the 6-month follow-up, participants are offered referrals if indicated, and SC participants can choose to receive IE or AST if they lack a meaningful response (defined as PTSD or alcohol use above study-entry criteria thresholds on structured interviews).

#### Retention

The project uses participant tracking, regular contacts, and incentives. Total possible compensation is US $277. This includes US $50 for intake, US $20 for the randomization visit, US $7 for rewards associated with the PRT, US $30 for the 1-month and 2-month follow-ups, US $10 per weekly self-report, a US $15 bonus for completing all weekly self-reports during either phase, and US $50 for the 6-month follow-up.

#### Interviewer Reliability

Assessors are trained to an 80% reliability standard. Interviews are audio recorded and interviewers use semistructured interviews to record their judgment of patient responses. After training, 10% of interviews are randomly selected to assess interrater reliability for PTSD (using PSS-I-5) and depression (using Quick Inventory of Depressive Symptomatology–Clinician Rated) severity, suicidality (using Columbia Suicide Severity Rating Scale), and comorbid diagnoses (using Structured Clinical Interview for DSM-5).

### Interventions

See Table 2 for a session-by-session description of interventions.

**Table 2.** Content of imaginal exposure and alcohol skills training interventions by session.

Intervention and session	Description
Imaginal exposure	
1	Overview of treatment, psychoeducation on common reactions to trauma, brief overview of treatment rationale, and review of the target trauma
2	In-depth rationale for exposure, discussion of subjective units of distress, first imaginal exposure (20‐30 minutes), and processing
3	Imaginal exposure and processing
4	Imaginal exposure and processing, memory recounting on the most currently distressing parts, called “hot spots”
5	Imaginal exposure and processing of hot spots
6	Final imaginal exposure, review of progress, discussion of maintaining gains, and termination of therapy
Alcohol skills training	
1	Review of the treatment rationale, values clarification activity, discussion of the client’s short-term goals that align with their values, and discussion of alternative rewarding activities
2	Discuss and practice generating an action plan for rewarding activities and identifying and addressing barriers to engaging in the action plan
3	Discuss and practice cognitive restructuring with an emphasis on addressing negative thinking that interferes with engaging in rewarding activities or contributes to problematic alcohol use
4	Discuss and practice increasing rewarding relationships through increasing the client’s social support and maintaining rewarding relationships
5	Discuss and practice setting long-term goals consistent with the participant’s values and reviewing how to identify and address barriers for long-term goals
6	Review the participant’s progress in treatment, encourage continued use of the skills learned, termination of therapy

#### IE

The IE protocol consists of six 50-minute manualized telehealth sessions delivered twice-weekly over 3 weeks. IE is based on the prolonged exposure manual [22] and includes psychoeducation, repeated imaginal exposure to the trauma memory, processing of the exposure and memory content, and consolidation of new learning. IE does not include in vivo exposure or homework. During IE, the client recounts the memory of the sexual assault, with the therapist probing for the client’s distress ratings approximately every 5 minutes. Processing includes praise, inquiries about the client’s experience, reflections on revisiting the trauma memory, and promoting adaptive meaning-making. This brief IE intervention has shown previous efficacy [23,53].

#### AST

The AST protocol [] (MA Bedard-Gilligan et al, unpublished data, 2020) consists of six 50-minute manualized telehealth sessions delivered twice-weekly over 3 weeks. AST is based on brief cognitive behavioral programs commonly used in alcohol treatment [54,55]. “At-home-practice” with accompanying worksheets is included. AST addresses reward deficits. It focuses on increasing alcohol-free rewarding activities and learning tools to address barriers and increase support around rewarding activities. Increasing alternative rewarding activities is presented as an approach to reducing the negative effects of drinking by finding more adaptive ways to experience rewards in life.

#### SC

SC consists of 12 20-minute phone sessions delivered twice-weekly for 6 weeks (MA Bedard-Gilligan and LA Zoellner, unpublished data, 2020). SC is a semistructured active control therapy and has demonstrated symptom reductions for trauma survivors [56]. The intervention combines psychoeducation, self-monitoring, directed writing outside sessions (ie, ~20 minutes about nontrauma-related stressors twice-weekly), and support. Sessions are not trauma-focused or alcohol-focused. Therapists are nondirective, ask open-ended questions, and provide validation. If the patient discloses assault-related or drinking-related stressors, the therapist uses supportive strategies, but does not instigate conversations about PTSD, assault, or alcohol use, and does not engage in any formal cognitive restructuring, exposure, or alcohol change strategies (eg, motivational interviewing).

### Therapist Training and Fidelity

Therapists are advanced doctoral students or clinical psychologists with training in PTSD and/or AUD. Over the course of the study, 18 therapists delivered the treatments. IE and AST sessions are recorded for supervision and fidelity coding. Therapists are trained (ie, session review and experiential practice) and all therapists participate in weekly supervision (ie, verbal case reports and reviewing of digital sessions). The initial training and ongoing supervision are intended to minimize drift and increase adherence to the protocol. Therapists provide only IE or AST at one time, although some therapists are trained to deliver both (ie, IE for 6 months and then AST for 6 months). A clinician with expertise in exposure and AST who is not affiliated with the study reviews 10% of the sessions and rates fidelity. SC sessions are not reviewed for fidelity and are not recorded.

### Ethical Considerations

The study protocol was approved by the University of Washington Institutional Review Board (STUDY00008021). Informed consent was obtained and participants were offered the option to opt out of the study and any of its procedures at any time. The potentially vulnerable sample requires thoughtful consideration of strategies to manage safety and clinical needs. The study has procedures to verify and record participant location if crisis services need to be dispatched and to confirm the confidentiality of the current location. All participants receive crisis line information at the start of the study and as needed. Staff have received training in procedures for managing risk, including assessing suicidal intent and plan via the Columbia Suicide Severity Rating Scale [45]. We use the Stanley Brown Safety Plan [57] when participants indicate potential acute risk. Participants in active treatment are monitored weekly for symptom exacerbation, including suicidal thoughts. Endorsement of suicidal thinking or behavior is addressed in clinical sessions with the study therapist to ensure safety and stability. For participants assigned to no treatment in phase 2, staff monitor weekly assessments, and the therapist from phase 1 initiates outreach if risk increases or changes. Procedures to protect confidentiality include anonymous study IDs, storing data on secure Health Insurance Portability and Accountability Act (HIPAA)–compliant servers, and a National Institutes of Health Certificate of Confidentiality. Participants may decline to answer questions for any reason or withdraw from the study at any time. Finally, the study has a data and safety monitoring board (DSMB) consisting of 4 members with expertise in alcohol use, PTSD, and sexual assault. The board reviews the study design, materials, data management, and participant procedures, meeting at least twice per year to review study progress, unexpected and adverse events, and protocol compliance. The DSMB is authorized to halt the study if adverse effects are observed.

### Analysis Plan

#### Preliminary Steps

As a first step in our analyses, we will calculate descriptive information on demographic characteristics, index traumatic event (ie, recent sexual assault), and trauma and treatment history by condition. We will also calculate means and standard deviations for all primary outcomes by condition and time point and calculate the prevalence of a 20% reduction in primary outcome measures at 1-month, 2-month, and 6-month follow-up. This descriptive information will be included in our outcome papers, making for straightforward use of study results in meta-analyses.

#### Handling of Missing Data

We will assess differences in completion rates for the 1-month, 2-month, and 6-month follow-up assessments using chi-square tests for contingency tables. Assessment of condition differences in completion of weekly self-report assessments will involve comparing the number of assessments completed and common patterns of attrition (eg, completion of all assessments, early dropout, late dropout, and intermittent missed assessments). Modeling of condition differences will use maximum likelihood estimation, with variances and covariances of model covariates estimated, or multiple imputation so that participants with partial data (eg, missed follow-ups) will be included with the assumption that data are missing at random (MAR) after accounting for nonmissing covariates [58]. This approach will reduce bias in model estimates due to missingness associated with condition assignment, baseline measures of PTSD symptom severity, alcohol use, and time since the traumatic event. We will also use pattern-mixture modeling to assess whether estimates of condition differences vary across different patterns of missingness and there is evidence of the MAR assumption not holding [59].

#### Multiple Comparisons

The study involves 3 conditions in phase 1 and 5 conditions in phase 2, and the aims call for multiple-condition comparisons at multiple time points on multiple outcomes. This can potentially lead to inflated type I errors when using a statistical significance criterion of *P*<.05. Although we will report *P* values, our primary guard against type I errors will be to present clear information on effect sizes and uncertainty of estimates. Condition differences will be expressed (1) on the metric of continuous outcomes and as standardized effects (*d*), (2) as percentage differences in counts for count outcomes, and (3) as prevalence differences for dichotomous outcomes. Estimates of uncertainty will be shown as confidence intervals. Another guard against overinterpreting chance findings will be to present condition differences in terms of a hierarchy of more general contrasts (eg, any assignments involving IE or AST vs SC) before considering more specific contrasts (eg, IE->AST vs AST->IE). In other words, the findings regarding more specific contrasts between any 2 of 5 conditions after phase 2 will be presented within the context of broader contrasts.

#### Aim 1 and Aim 2: Condition Differences at 1-Month, 2-Month, and 6-Month Follow-Ups

Analyses will follow an intent-to-treat (ITT) approach, based on data from all randomized participants, regardless of treatment completion. Analyses will provide estimates of condition differences at follow-up time points, adjusted for covariates, with the representation of uncertainty in these estimates provided by confidence intervals. At each follow-up assessment, the primary assessment of condition differences in PTSD severity (ie, PSS-I-5) and alcohol use (ie, TLFB) will be assessed with regression models that control for preintervention baseline PSSI-5-I scale score, baseline TLFB measure of past-month drinking days, and months since assault. Assessing condition differences at each of the 3 follow-up time points addresses analytically different questions, with differences at 1-month and 2-month follow-ups capturing immediate postintervention effects on PTSD symptom severity and alcohol use and differences at 6-month follow-up showing whether differences are maintained multiple months after completing the intervention phase. Assessing differences controlling for baseline provides more precise estimates of condition differences than using change scores [60]. For models with average drinks consumed on drinking days as the outcome, intake scores on that measure will also be included as a covariate. Estimated effects of indicator-coded condition assignment will be used to assess condition differences [61].

At 1-month follow-up, we will address aim 1 with contrasts between (1) both IE and AST and SC and (2) IE and AST. At 2-month and 6-month follow-ups, we will examine contrasts between all conditions involving IE or AST (alone or in combination) and SC. We will address Aim 2 by contrasting (1) IE alone or AST alone with conditions involving both IE and AST and (2) conditions involving both IE and AST, comparing by which intervention is first.

Additionally, as an illustration of condition differences, we will use multilevel growth models [62] to compare conditions involving IE or AST with SC in terms of change across the 4 time points (aim 1). Spline models will include two specifications of time to represent (1) change across the active intervention period between baseline and 2-month follow-up, when the conditions involving IE and AST are hypothesized to improve relative to SC; and (2) change between 2-month and 6-month follow-ups, when improvements that occurred during the active intervention are hypothesized to be maintained. Results will be shown graphically in terms of marginal expected values by condition and time point, adjusting for model covariates. PTSD severity and average drinks consumed on drinking days are expected to have approximately normal distributions, and linear models will be used. Condition effect estimates from linear models have a straightforward interpretation as differences on the metric of the dependent variable. We will also calculate standardized effects (*d*) based on the unstandardized effect estimates divided by the baseline SD of the given outcome. Generalized linear models with a negative binomial distributional specification will be used for the count measure of drinking days. Effects for drinking days will be expressed as count ratios, interpretable as the percentage difference in drinking days associated with the condition [63]. For growth models, marginal estimates will provide model-expected values of outcomes at each time point by condition adjusted for covariates.

#### Growth Models of Weekly Self-Report Data Through 2-Month Follow-Up

We will use multilevel growth models to analyze data from weekly self-administered measures of PTSD, drinking, alcohol cravings, and fear and reward collected through the 2-month follow-up. This will involve 9 time points of data: baseline followed by 6 weekly assessments and 2 follow-ups. The models will include specifications of time to represent the weekly rate of change between baseline and 1-month follow-up (phase 1), followed by the rate of change between the 1-month and 2-month postbaseline follow-ups (phase 2). A similar hierarchy of contrasts will be assessed, with IE and AST vs SC being of primary interest in differences in change in phase 1 and conditions in either IE or AST vs SC being of interest in phase 2.

#### Aim 3: Condition Differences by Potential Moderators

Exploratory analyses addressing aim 3 will examine recovery and intervention effects based on baseline performance on fear (ie, AX+/BX–) and reward (ie, PRT) tasks and time since assault. Each of these variables will be added to the regression models described in the *Aim 1 and Aim 2: Condition Differences at 1-Month, 2-Month, and 6-Month Follow-Ups* section, first to assess main effects on recovery (ie, as reflected in decrease in PTSD symptoms and alcohol use) and second, to assess condition differences at different levels of these potential moderators. Estimates of intervention effects across scores on the fear and reward tasks and at different lengths of time since assault will be generated from models with interaction terms (ie, condition by moderators).

#### Power

Based on findings from prior clinical trials [64], we expected medium-to-large intervention effects. Accordingly, the design called for a total sample size of 180. Primary power analyses were based on ANOVA models using the power calculator in Stata 15.0 (StataCorp LLC). Accounting for 25% attrition between baseline and follow-up, an enrolled sample of 180 provides 0.8 power with an α of .05 to detect a *d* of 0.6 for differences between any 2 of the initial 3 conditions (ie, aim 1 hypotheses). For aim 2 analyses, where some condition-defined contrasts involve groups half the size of the initial groups (eg, IE or AST only), contrasts between these smaller groups have 0.8 power to detect a *d* of 0.86. Precision of estimates will be increased by including baseline scores of outcomes as covariates and estimation that allows for the inclusion of cases with partial data.

## Results

The study was funded in September 2019. Due to delays associated with the COVID-19 pandemic, recruitment began in March 2021 and ended in December 2025. Enrollment into the study fell below expectations (expected sample=180; final enrolled sample=82). Through active recruitment, 629 individuals were screened and 326 were scheduled for a baseline intake assessment. Primary reasons for exclusion at screening were not having experienced a sexual assault in the eligibility time frame (4 weeks to 1 year) and PTSD or alcohol use being below the eligibility threshold. The primary reasons for not being eligible at intake were canceling or not showing up to the appointment (we completed 210 of 326 scheduled intakes), subthreshold PTSD or alcohol use, or having an exclusionary diagnosis or clinical rule-out (eg, self-harm or suicidality). Retention at follow-ups was high: 85% at 1-month follow-up, 76% at 2-month follow-up, and 77% at 6-month follow-up. Data analysis and publication of primary outcomes are expected by the summer of 2027.

Because recruitment fell below expectations, the uncertainty of estimates will be larger, and power will be lower than planned. Considering this, and as noted above, we will heed concerns about overreliance on significance testing [65,66] and provide a clear representation of both estimated effect sizes and the uncertainty of those estimates. Rather than making strong inferences about the efficacy of different treatment types based on *P* values, emphasis will be placed on providing a clear and straightforward presentation of results that can be used in future meta-analyses.

## Discussion

Project SARAH explores shared vulnerabilities of PTSD and alcohol misuse as predictors of treatment response and assesses the effects of different interventions. The trial is an example of bench-to-bedside science, integrating understanding of fear and reward processes with clinical questions around how to best promote recovery following trauma exposure. The trial will help us develop targeted, personalized, and effective interventions. At the 1-month follow-up, we expect that both IE and AST will outperform SC on PTSD and drinking outcomes and that IE will lead to greater decreases in PTSD and fear and AST to greater decreases in alcohol use and greater increases in reward, consistent with the theorized mechanisms of the interventions. At the 2-month follow-up, we expect that individuals who receive both active interventions (IE and AST) will show greater improvements than those who receive only 1 intervention (IE or AST) and those who receive SC. Our exploratory aims are to predict outcomes based on baseline impairments in fear and reward (self-report measures and behavioral tasks) and we expect greater fear impairments to predict greater PTSD at final follow-up and greater impairments with reward will predict greater alcohol use at final follow-up.

Making interventions briefer and more accessible is necessary for widespread dissemination of evidence-based treatments. By exploring fear and reward processes, we focus on shared vulnerabilities that are viable clinical targets. Interventions include IE, which directly promotes fear extinction, and AST, which addresses deficits in reward processes. Most alcohol strategies in the context of PTSD target coping motives (ie, reducing alcohol to cope with distress). AST addresses both coping and enhancement (ie, reducing alcohol to experience reward) motives; since PTSD is associated with known reward deficits, enhancement motives may also be conceptualized as coping-oriented (ie, using alcohol to increase rewards that are missing). The importance of enhancement motives is often overlooked in studies and treatment for PTSD and alcohol misuse that do not address reward processes.

The doubly randomized design will make it possible to assess whether targeting one or both mechanisms is needed to achieve clinically meaningful gains. Several protocols exist that target PTSD and alcohol or substance misuse concurrently, but they require substantial engagement (eg, multiple sessions and homework) and have high dropout rates [67,68]. Lower dose options (eg, 6 sessions or less, ≤3-week duration) have also been found to be effective for PTSD [23], but it is not known if they are effective for concurrent PTSD and alcohol misuse. In the initial months after sexual assault, targeting only one vulnerability may be enough to “move the needle,” helping clients learn skills and modify behaviors that put them back on a natural recovery trajectory; indeed, effective early interventions do tend to be briefer than those designed for chronic trauma-related symptoms [12]. This study will help elucidate whether briefer, better targeted treatments are effective, and if so, for whom.

The COVID-19 pandemic necessitated substantial changes to study protocols. Fortunately, research supported the delivery of trauma-focused treatments via telehealth [69,70], which enabled us to make this transition with limited concerns for safety, acceptability, or feasibility. Delivering the interventions remotely enabled recruitment across Washington and California, which will result in a more diverse sample. The changes did, however, also impact safety protocols, data collection methods, and outcome variables (eg, no psychophysiological indicators on the AX+/BX- task). These changes set back the study timeline and resulted in a smaller-than-intended sample. Notably, it is challenging to engage survivors in services soon after trauma, even without the context of a pandemic and clinical practice lacks a gold-standard treatment for the acute recovery phase [12]. This is unfortunate, as the year after trauma is a window when the potential for recovery is greatest.

There are several limitations to the study. Notably, and most prominently, our recruitment fell below expectations. Despite our considerable efforts, recruiting recent sexual assault survivors who met both our PTSD and alcohol use criteria was challenging. Future studies should consider study designs that do not rely on community recruitment but instead use methods such as embedding study procedures in existing service clinics, preplanned partnerships with organizations that serve acute survivors, and/or leveraging emergency service use to directly identify potential participants (eg, accessing electronic medical records). An additional limitation is our inability to look directly at therapist effects given the relatively small number of patients assigned to each therapist. Thus, it is not possible to account for therapist effects in the analyses of condition differences, which may be a threat to the validity of the findings [71]. The study, which was designed to be a mechanism-focused efficacy trial, did not assess the acceptability and feasibility of the interventions, which could inform future dissemination and implementation efforts.

Strengths of the study include its novel phased design, the incorporation of behavioral tasks and self-report measures to assess mechanisms, and robust methods around assessment and intervention delivery. This study tested brief, accessible interventions that target theorized mechanisms to improve both PTSD and alcohol misuse acutely. Project SARAH will provide crucial information on treatment targets and comparative efficacy of intervention strategies.

## Supplementary material

10.2196/107568 Checklist 1 SPIRIT 2025 checklist.

10.2196/107568 Peer Review Report 1 Peer review report by the National Institute on Alcohol Abuse and Alcoholism (NIAAA R01AA027499).

## Abbreviations

AST: alcohol skills training
AUD: alcohol use disorder
DDQ: Daily Drinking Questionnaire
*DSM-5*: *Diagnostic and Statistical Manual of Mental Disorders* (Fifth Edition)
DSMB: data and safety monitoring board
HED: heavy episodic drinking
HIPAA: Health Insurance Portability and Accountability Act
IE: imaginal exposure
ITT: intent-to-treat
MAR: missing at random
PRT: Probabilistic Reward Task
PSS-I: PTSD Symptom Scale–Interview
PSS-I-5: PTSD Symptom Scale–Interview for DSM-5
PSS-SR-5: PTSD Symptom Scale-–Self-Report
PTSD: posttraumatic stress disorder
SARAH: Sexual Assault Recovery and Hope
SC: supportive counseling
SPIRIT: Standard Protocol Items: Recommendations for Interventional Trials
TLFB: Timeline Followback
US: unconditioned stimulus
