# Supplementary material for: Acute Interventions for Posttraumatic Stress Disorder and Alcohol Misuse in Women Exposed to Sexual Assault: Protocol for a Phased Randomization Trial (Project SARAH)
Source: JMIR Res Protoc. 2026 Nov 3;15:e107568. doi: 10.2196/107568 (PMC13730508; doi:10.2196/107568)
Supplement: Supplementary file 1 [file resprot-v15-e107568-s002.pdf]

SUMMARY STATEMENT
(Privileged Communication)

PROGRAM CONTACT:
Dr. Deidra Roach
301-443-5820
droach@mail.nih.gov

Release Date: 07/01/2019
Revised Date:

Application Number: **1 R01 AA027499-01A1**

Principal Investigator

BEDARD-GILLIGAN, MICHELE A

Applicant Organization: UNIVERSITY OF WASHINGTON

Review Group:	AA-3<br>Clinical, Treatment and Health Services Research Review Subcommittee
Meeting Date:	06/21/2019
Council:	OCT 2019
Requested Start:	09/01/2019
RFA/PA:	PA19-055
PCC:	AC D U

Project Title:	Understanding and testing recovery processes for PTSD and alcohol use following sexual assault
SRG Action:	Impact Score:25 Percentile:9 #
Next Steps:	Visit https://grants.nih.gov/grants/next_steps.htm
Human Subjects:	30-Human subjects involved - Certified, no SRG concerns
Animal Subjects:	10-No live vertebrate animals involved for competing appl.
Gender:	2A-Only women, scientifically acceptable
Minority:	1A-Minorities and non-minorities, scientifically acceptable
Age:	7A-Only Adults, scientifically acceptable

Project Year	Direct Costs Requested	Estimated Total Cost
1	395,349	540,877
2	403,521	552,058
3	412,250	564,000
4	455,770	623,539
5	454,280	621,501
TOTAL	2,121,170	2,901,975

ADMINISTRATIVE BUDGET NOTE: The budget shown is the requested budget and has not been adjusted to reflect any recommendations made by reviewers. If an award is planned, the costs will be calculated by Institute grants management staff based on the recommendations outlined below in the COMMITTEE BUDGET RECOMMENDATIONS section.
EARLY STAGE INVESTIGATOR
NEW INVESTIGATOR

1R01AA027499-01A1 BEDARD-GILLIGAN, MICHELE

EARLY STAGE INVESTIGATOR

RESUME AND SUMMARY OF DISCUSSION: This is an amended R01 application by an Early Stage Investigator proposing a doubly randomized clinical trial to examine two key cross-cutting mechanisms (impaired fear extinction and reward dysfunction) as treatment targets among sexual assault survivors with alcohol and PTSD symptoms. Proposed studies are highly significant and focus on an important and understudied group. The study design is innovative and the central hypothesis that decreasing fear should increase rewards and increasing rewarding activities should decrease pathological fear and avoidance behavior is sound and well supported by preliminary data. The revised submission is highly responsive to prior critiques and has satisfactorily addressed most design issues. The research team is well-qualified and has been strengthened by the addition of Dr. Stappenbeck to complement the analysis team. A few minor concerns remain, but do not deter the overall impact of the proposed studies. Overall, there was an excellent to outstanding enthusiasm for this application.

DESCRIPTION: Following sexual assault, many individuals will develop chronic problems including posttraumatic stress disorder (PTSD) and alcohol use disorders (AUD). Intervention provided soon after assault can decrease the risk of developing chronic psychopathology and associated negative consequences. Interventions that address common underlying mechanisms of PTSD and alcohol use, such as fear and reward systems, have strong potential utility as efficacious and accessible interventions for clinicians treating patients recently exposed to sexual assault. This proposal is designed to test fear and reward as crucial processes underlying recovery following sexual assault and elucidate the most efficacious treatment targets. Employing experimental tasks (safety-signal learning paradigm and probabilistic reward task) to capture baseline underlying vulnerabilities in fear and reward systems respectively will allow for exploration of how these processes impact recovery. A randomized clinical trial (N = 180) will be conducted to test efficacy of intervention approaches that target PTSD or alcohol use compared to supportive telehealth. In addition, a phased study design will allow for exploration of efficacy of primary and secondary intervention approaches to test the questions of 1) whether it is more efficacious to target PTSD or alcohol use first; and 2) whether it is necessary to target both PTSD and alcohol use to facilitate recovery or if one is sufficient. This proposal is significant in exploring transdiagnostic mechanisms implicated in recovery following sexual assault, fear and reward, and using a novel design to compare efficacy, ordering, and necessity of two distinct intervention approaches.

PUBLIC HEALTH RELEVANCE: Sexual assault can lead to devastating consequences including the development of chronic conditions including posttraumatic stress disorder (PTSD) and alcohol use disorders (AUD). Interventions delivered soon after exposure to assault can decrease the long-term negative consequences of sexual assault but existing interventions are limited in their ability to target concurrent PTSD symptoms and alcohol use and little is known about how to make best practice treatment decisions in the early period following sexual assault. A greater emphasis on transdiagnostic processes that are related to both PTSD and alcohol use, such as fear and reward systems, can elucidate mechanisms of recovery, lead to the development of more effective intervention approaches, and guide clinical decision making for patients recently exposed to sexual assault.

CRITIQUE 1

Significance: 2
Investigator(s): 1
Innovation: 3
Approach: 3
Environment: 1

Overall Impact:

This revised R01 proposal by an early stage investigator is designed to test fear and reward as crucial processes underlying both natural and therapeutic recovery following sexual assault. Using a doubly randomized clinical trial design, this trial tests the efficacy of a brief imaginal exposure or alcohol skills training intervention to prevent the development of chronic PTSD and AUD. Participants will be 180 women who have been sexually assaulted in the previous month and report elevated PTSD symptoms and alcohol misuse. Fear (inhibitory learning) and reward deficits (probabilistic reward) will be examined prior to randomization using well-established paradigms examining impaired reward functioning and inhibitory fear learning. Participants will be randomly assigned to imaginal exposure (n = 60, 6 sessions over 3 weeks), alcohol skills training (n = 60, 6 sessions over 3 weeks), or a supportive telehealth condition (n = 60; 12 session over 6 weeks). Following the initial intervention, participants in the active intervention conditions will be re-randomized to either the alternative intervention or to no further intervention. Self-report measures of fear and reward functioning administered weekly will examine intervention-related changes in these transdiagnostic constructs. Key outcomes include alcohol use and PTSD symptoms assessed at 2-, 3- and 6-month follow-ups. Strengths of this proposal include the strong team and research environment, the examination of an important and understudied group (sexual assault survivors with alcohol and PTSD symptoms), the innovative doubly randomized design that examines two key potentially cross-cutting mechanisms (impaired fear extinction and reward dysfunction) as treatment targets. The hypothesis that decreasing fear should increase rewards and Increasing rewarding activities should decrease pathological fear and avoidance behavior is well supported. Investigators were highly responsive to initial reviews. Limitations include the short follow-up period, the fact that the alcohol intervention may not be appropriate for the wide range of drinking in the anticipated sample (from college student binge drinkers to adults with chronic AUD), the lack of clinical/predictive validity data for the reward probability task, and the fact that integrated interventions approaches have been developed for PTSD treatment and prevention by this group. Additionally, this team is currently testing a similar integrated treatment for sexual assault prevention in a funded R34 grant so there is some concern about overlap. Overall, however, this is an innovative and potentially significant proposal from a very strong investigative team.

1. Significance:

Strengths

- Sexual assault can lead to devastating consequences including the development of chronic conditions including posttraumatic stress disorder (PTSD) and alcohol use disorders (AUD).
- A greater emphasis on “transdiagnostic” processes that are related to both PTSD and alcohol use, such as fear and reward systems, may elucidate mechanisms of recovery, and lead to the development of more effective intervention approaches.
- Several studies have examined interventions delivered in the weeks/months after trauma (acute interventions), and most found positive results, but no studies have looked at efficacy of alcohol interventions as stand-alone interventions in the acute recovery phase following sexual assault.
- Sexual assault is associated with less response to PTSD/AUD treatment, compared to other trauma types. This supports the significance of enhancing treatments for victims of sexual assault
- Identifying basic, personalized mechanisms that predict recovery trajectory for both PTSD and alcohol use and that predict response to early intervention could have clinical significance
- Only 9% of people seek treatment within the year after trauma exposure, and less than 20% seek treatment for AUD in the first year of the disorder, so intervening prior to help-seeking is key

Weaknesses

- Although there are no published studies on comparable efficacy of targeting PTSD or alcohol use acutely following trauma exposure, several studies suggest that PTSD treatments that are effective for sexual assault victims, and there is fairly widespread consensus at this point in the PTSD/AUD comorbidity treatment literature that integrated treatments are preferred to sequential treatments.
- The assumption is that AUD begins with the sexual assault but for many participants AUD may be chronic, yet AUD severity is not tested as a moderator.

2. Investigator(s):

Strengths

- The PI is an early stage investigator and Assistant Professor in the University of Washington Department of Psychiatry and Behavioral Sciences who is currently the principal investigator of two grant funded research projects on treatments for PTSD and substance misuse (R34AA022966, R34DA034529), focused on alcohol and cannabis use specifically.
- The team has a record of collaborations and all the requisite skills necessary to complete this study and disseminate the results. The Co-Is are established leaders in the field of alcohol use and trauma and have a history of successful collaborations.
- Co-I Fleming provides statistical expertise and consultant Gallop will provide specific expertise in multiple randomization designs.

Weaknesses

- None noted.

3. Innovation:

Strengths

- PTSD and AUD are linked to deficits in processing fear and reward and targeting one, particularly before chronic psychopathology develops, may have downstream cascade effects on decreasing the other. However, there are no human studies examining fear and reward processes in the aftermath of trauma exposure, despite the potential of better targeting these processes to promote recovery.
- The shared mechanism approach, focusing on fear and reward, and phased, doubly randomized clinical trial design is novel. However, there is a lack of data connecting the probabilistic reward measure to clinically relevant outcomes.

Weaknesses

- The individual intervention elements are not innovative, and they have been previously combined in integrated treatments and prevention for PTSD populations (including sexual assault victims) by this team.
- Additionally, previous research has investigated acute interventions for trauma, including imaginal exposure, and found moderate effect sizes. Studies have also examined brief alcohol interventions immediately following trauma. The authors note that both PTSD and substance use treatment approaches delivered separately appear effective for co-morbid patients, with both significantly decreasing both disorders. Finally, this group has another ongoing R34 testing an integrated brief (single session) cognitive intervention for PTSD and alcohol use among sexual assault survivors. It would seem ideal to have the results of that project inform the development of the proposed intervention.

4. Approach:

Strengths

- The study is a 3-arm randomized controlled trial (imaginal exposure, alcohol skills training, monitoring only) with a second randomization for intervention participants to either the alternative intervention or no additional intervention. This is an elegant study design that will allow for testing effects of the interventions in isolation and combined together.
- The chosen interventions are evidence-based and the relatively brief versions of these interventions increase potential for dissemination and address the issue of high dropout associated with more intensive treatments for chronic PTSD/AUD. Six sessions may be a good dose given the heterogeneity of the sample (ranging from young adult binge drinking to women with chronic AUD) although the intervention will still be perceived as too intense for some (e.g., young adult binge drinkers) and not intense enough for others (adults with chronic AUD).
- The authors make a compelling theoretical case that fear-avoid, and reward-approach systems may work in synchrony to direct behavior adaptively, with PTSD/AUD reflecting a dysregulated, imbalanced fear-reward system. The hypothesis that decreasing fear should increase rewards and Increasing rewarding activities should decrease pathological fear and avoidance behavior is well supported.
- The safety signal learning paradigm will be used to examine discriminative conditioning and fear extinction. Fear potentiated startle will be operationalized as an eyeblink response that will be measured via electromyography (EMG). The team's previous research suggests that safety signal learning deficits were strongly associated with higher pre-treatment PTSD hyperarousal symptoms and greater between-session extinction during imaginal exposure, showing that this paradigm taps into extinction processes predictive of therapeutic change.
- The team has relevant experience with the interventions, both of which they have found to be effective in other trauma samples. Alcohol skills training has been shown to reduce alcohol misuse and improve trauma symptoms
- Planned analyses with generalized linear models with growth modeling are appropriate.
- The team has experience recruiting a similar sample

Weaknesses

- The inclusion of EEG based Probabilistic Reward Task is a strength, but there is no evidence that these measures are reliable, sensitive to an alcohol-skills training intervention approach, correspond with measurement of substance-free reward experience in the environment, or predictive of alcohol-related outcomes. The fact that they are modulated by dopamine activity does not attest to their clinical/predictive validity.
- Impaired fear conditioning is a reasonable mechanism for AUD that develops following trauma, but many participants in the proposed study may drink for other reasons including social facilitation, thus reducing the relevance of the exposure therapy.
- Is treatment and prevention functionally different with this clinical picture? Treatment elements don't seem to be markedly different other than, to a degree, their brevity.
- The authors expanded the eligibility in response to reviews but do not directly address how the alcohol treatment elements will be relevant to the range of drinking they will likely enroll (from young adult occasional binge drinkers with little motivation to change to women with chronic and severe AUD). The assumption seems to be that drinking is stemming from the assault but for many young adults drinking may be tied to social facilitation, and for older women with more severe AUD the drinking may have preceded the assault.

- The RAPI is a measure that has been validated for measuring alcohol problems in teens/young adults and is not appropriate for general or older adult samples.

5. Environment:

Strengths

- The scientific environment at the University of Washington (UW) Department of Psychiatry and Behavioral Sciences is exceptional, including the Center for the Study of Health and Risk Behaviors (CSHRB) and Trauma Recovery Innovations (TRI) which are jointly housed in the Department.
- A Biopac MP150 system with transducers for electromyography (EMG), skin conductance response and level (SCR/SCL), respiration, and electrocardiography (ECG) is available for use. An air puff apparatus is in place for the safety-signal learning paradigm.

Weaknesses

- None noted.

Study Timeline:

Strengths

- Timeline is clear and appears to be reasonable

Weaknesses

- None noted.

Protections for Human Subjects:

Acceptable Risks and/or Adequate Protections

- the authors added a supportive counseling phone intervention as a control condition (instead of an assessment-only condition) which reduced participant risk

Data and Safety Monitoring Plan (Applicable for Clinical Trials Only):

Acceptable

- o The project will follow IRB and NIH policies regarding the monitoring and reporting of adverse events. Data will be stored on secured servers. The project will report to a DSMB.

Inclusion Plans:

- Sex/Gender: Distribution justified scientifically
- Race/Ethnicity: Distribution justified scientifically
- For NIH-Defined Phase III trials, Plans for valid design and analysis: Scientifically acceptable
- Inclusion/Exclusion Based on Age: Distribution justified scientifically
- Participants characteristics are consistent with the nature of the high risk sexual assault population. The focus is on women based on their higher risk of assault. There is a detailed plan for ensuring adequate recruitment of minority individuals (anticipated 30%).

Revision:

- The investigators were largely responsive to the initial critiques. To address concerns about feasibility of intervention in the first week's post-assault, the investigators expanded inclusion criteria to sexual assault occurring within the last year (1 month to 12 months post assault. This improves feasibility of recruitment.
- To address concerns that alcohol skills training does not exclusively focus on reward and that the probabilistic reward task (PRT) may not correspond to substance free reward functioning, the investigators increased the focus on behavioral activation in the intervention.
- To address concerns about the use of an assessment control condition, the investigators changed to a supportive telehealth intervention used in past studies with trauma-exposed patients. They have also added weekly measures of fear and reward.

Resource Sharing Plans:

Acceptable

- Data will be shared via the National Database for Clinical Trials.

Budget and Period of Support:

Recommend as Requested

CRITIQUE 2

Significance: 1
Investigator(s): 1
Innovation: 2
Approach: 2
Environment: 1

Overall Impact:

This revised application addressed the significant topic of risk for post traumatic stress disorder (PTSD) and alcohol use disorder (AUD) in women who experience a sexual assault.The applicant was responsive to the concerns raised in the review of the original proposal. Inclusion criteria were expanded to the experience of sexual assault 1 month to 12 months post assult to capture a time period that is early but not confined to the period of sharp trajectories of natural recovery. Design elements driven by this application's focus on prevention of psychopthology compared to treatment of chronic psychopathology were clarified. The revised application also increased focus on behavioral activation in the alcohol skills training to sharpen alignment with the hypothesized reward mechanism focus. The assessment control condition was replaced with an evidence-based telehealth intervention to control for non-specific treatment factors and address human subjects' concerns. Weekly fear and reward measures were added to enhance process/moderator analyses, although the relation of these new self-report measures to performance assessed in the fear and reward tasks was not clear. The assessment of drinking behaviors was expanded, and heavy drinking was added as an outcome, although the new drinking consequence measure is most appropriate for younger aged samples. Support letters from large sexual assault service organizations in the area increased confidence in the feasibility of recruitment. The investigative team, including the addition of Dr. Stappenbeck to compliment the analysis team, is ideally suited to the proposed work and the environment is highly supportive. Overall, this application was substantially strengthened, although some outstanding questions remained regarding new assessments.

1. Significance:

Strengths

- Targeted focus on women who experienced sexual assault - a potent precipitant of PTSD, AUD, and treatment resistance.
- Examination of early intervention effects has promise for interrupting the development of chronic treatment-resistant disorders.
- Attention to critical ‘how’ and ‘for whom’ questions in studying treatment benefit.
- Inclusion of the exploratory aim to link vulnerabilities in fear and reward processing to imaginal exposure and alcohol skills training that includes behavioral activation.

Weaknesses

- None noted.

2. Investigator(s):

Strengths

- The PI is an early stage investigator with two R34 Awards from NIDA and NIAAA focused on PTSD and substance use and is a licensed psychologist.
- Dr. Stappenbeck was added to the investigative team to add depth to the biostatistics expertise
- Overall, this team of investigators has a strong collaborative history and is well equipped to complete the proposed work.

Weaknesses

- None noted.

3. Innovation:

Strengths

- The focus on early/preventive interventions to interrupt the progression of chronic psychopathology is novel in the AUD/PTSD area
- The exploration of fear and reward processing as underlying processes that affect recovery and intervention effects following sexual assault.
- The doubly randomized design will allow individual and sequential/combined analysis of treatment effects

Weaknesses

- None noted.

4. Approach:

Strengths

- The interventions to be employed are evidence-based and feasible. The use of an active control condition, supportive telehealth, added strength.
- Feasibility of recruitment increased through two referral sources that provided letters of support.
- The safety signal learning paradigm and the probabilistic reward task to assess fear and reward remained strengths.
- Alcohol risk inclusion now based on binge drinking behavior rather than negative consequences of use that may take more time to emerge.

Weaknesses

- The relation of the constructs measured by the reward and fear self-report measures to behavior/psychophysiology in the safety signal learning paradigm and the probabilistic reward task was not clear.
- Inclusion criteria stated, “Reports a sexual assault in the last 4 weeks to 1 year.” It was not entirely clear but appeared to mean those reporting in past month will be excluded.
- The Rutgers Alcohol Problem Index is geared towards adolescent and young adults and may not be a good choice for the heterogeneous age groups in the study including those up to age 65.

5. Environment:

Strengths

- The research environment at the University of Washington is strong and supportive including Centers dedicated to the study of trauma, health, and risk.

Weaknesses

- None noted.

Study Timeline:

Strengths

- The timeline appeared appropriate to accomplish the proposed work.

Weaknesses

- None noted.

Protections for Human Subjects:

Acceptable Risks and/or Adequate Protections

- Safeguards are in place to minimize risks due to psychological distress from study procedures and breach of confidentiality.
- Referral are available additional services if needed and for crisis intervention.

Data and Safety Monitoring Plan (Applicable for Clinical Trials Only):

Acceptable

- Data will be monitored for high risk responses (e.g., suicidality).
- Institutional and NIH policies will be followed for managing and reporting adverse events.
- A Data Safety and Monitoring Board will be established for overview.

Inclusion Plans:

- Sex/Gender: Distribution justified scientifically
- Race/Ethnicity: Distribution justified scientifically
- For NIH-Defined Phase III trials, Plans for valid design and analysis: Not applicable
- Inclusion/Exclusion Based on Age: Distribution justified scientifically
  - Women only included as they are disproportionately affected by sexual assault and most victims are women. 35% non-Caucasians are targeted; a plan is in place to support minority recruitment. Children less than 18 years are not included due to difference in

developmental processes from adults; older than 65 excluded due to low representation in the population of women experiencing recent sexual assault.

Resubmission:

- Overall, the applicant was responsive to the concerns raised in the review of the original application.
- Inclusion criteria were expanded to the experience of sexual assault 1 month to 12 months post assault to capture a time period that is early but not confined to the period of sharp trajectories of natural recovery.
- Clarification of design elements driven by this application's focus on prevention of psychopathology compared to treatment of chronic psychopathology.
- Addition of Dr. Stappenbeck to compliment the analysis team.
- Increased focus on behavioral activation in the alcohol skills training to sharpen alignment with the reward mechanism focus
- Replacement of the assessment control condition with a supportive telehealth intervention of demonstrated efficacy to control for non-specific treatment factors and address human subjects' concerns.
- Addition of weekly fear and reward measures to enhance process/moderator analyses.
- Expanded assessment of drinking behaviors and addition of heavy drinking as an outcome.
- Added support letters from large sexual assault service organizations to boost feasibility of recruitment.

Resource Sharing Plans:

Acceptable

Budget and Period of Support:

Recommend as Requested

CRITIQUE 3

Significance: 2
Investigator(s): 2
Innovation: 2
Approach: 3
Environment: 2

Overall Impact:

This is a revised R01 application to conduct a randomized controlled trial testing imaginal exposure for trauma and CBT for alcohol use among women who experienced sexual assault in the prior month. The original application was deemed overly ambitious based on the complex design and lack of statistical power. Insufficient justification for the early intervention (within 1 month of assault) was raised as a threat to generalizability and inclusion criteria requiring 2+ alcohol problems was deemed restrictive by reviewer #1. The transdiagnostic focus was not fulfilled by the use of the reward and fear tasks, which are rather exploratory. Several changes were made in response to the original critiques, including a broader inclusion criterion (assault in the past year), changes to the control condition, and additional repeated assessments to boost statistical power. Feasibility issues were addressed with letters of support from sexual assault organizations. While the application was improved and the team is

excellent, the response to the significance section was not particularly compelling and the changes made are moderate (e.g., study design and sample size remained unchanged). Preliminary data from studies led by the PI would have been desirable as well. Nonetheless, this is a strong team and the proposal is well constructed with the potential to be impactful.

Protections for Human Subjects:

Acceptable Risks and/or Adequate Protections

Data and Safety Monitoring Plan (Applicable for Clinical Trials Only):

Acceptable

Inclusion Plans:

- Sex/Gender: Distribution justified scientifically
- Race/Ethnicity: Distribution justified scientifically
- For NIH-Defined Phase III trials, Plans for valid design and analysis: Scientifically acceptable
- Inclusion/Exclusion Based on Age: Distribution justified scientifically

Budget and Period of Support:

Recommend as Requested

THE FOLLOWING SECTIONS WERE PREPARED BY THE SCIENTIFIC REVIEW OFFICER TO SUMMARIZE THE OUTCOME OF DISCUSSIONS OF THE REVIEW COMMITTEE, OR REVIEWERS' WRITTEN CRITIQUES, ON THE FOLLOWING ISSUES:

PROTECTION OF HUMAN SUBJECTS: ACCEPTABLE

INCLUSION OF WOMEN PLAN: ACCEPTABLE

INCLUSION OF MINORITIES PLAN: ACCEPTABLE

INCLUSION OF CHILDREN PLAN: ACCEPTABLE

COMMITTEE BUDGET RECOMMENDATIONS: The budget was recommended as requested.

Footnotes for 1 R01 AA027499-01A1; PI Name: BEDARD-Gilligan, MICHELE A

Ad hoc or special section application percentiled against "Total CSR" base.

NIH has modified its policy regarding the receipt of resubmissions (amended applications). See Guide Notice NOT-OD-14-074 at http://grants.nih.gov/grants/guide/notice-files/NOT-OD-14-074.html. The impact/priority score is calculated after discussion of an application by averaging the overall scores (1-9) given by all voting reviewers on the committee and multiplying by 10. The criterion scores are submitted prior to the meeting by the individual

reviewers assigned to an application, and are not discussed specifically at the review meeting or calculated into the overall impact score. Some applications also receive a percentile ranking. For details on the review process, see http://grants.nih.gov/grants/peer_review_process.htm#scoring.

MEETING ROSTER

Clinical, Treatment and Health Services Research Review Subcommittee
National Institute on Alcohol Abuse and Alcoholism Initial Review Group
NATIONAL INSTITUTE ON ALCOHOL ABUSE AND ALCOHOLISM
AA-3
06/21/2019

Notice of NIH Policy to All Applicants: Meeting rosters are provided for information purposes only. Applicant investigators and institutional officials must not communicate directly with study section members about an application before or after the review. Failure to observe this policy will create a serious breach of integrity in the peer review process, and may lead to actions outlined in NOT-OD-14-073 at https://grants.nih.gov/grants/guide/notice-files/NOT-OD-14-073.html and NOT-OD-15-106 at https://grants.nih.gov/grants/guide/notice-files/NOT-OD-15-106.html, including removal of the application from immediate review.

CHAIRPERSON(S)

MIRANDA, ROBERT JR., PHD
ASSOCIATE PROFESSOR
DEPARTMENT OF PSYCHIATRY & HUMAN BEHAVIOR
CENTER FOR ALCOHOL & ADDICTION STUDIES
BROWN UNIVERSITY
PROVIDENCE, RI 02906

MEMBERS

ARIAS, ALBERT JOSEPH, MD
ASSOCIATE PROFESSOR
DEPARTMENT OF PSYCHIATRY
DIVISION OF ADDICTION PSYCHIATRY
VIRGINIA COMMONWEALTH UNIVERSITY
RICHMOND, VA 23284

BATES, MARSHA E., PHD
DISTINGUISHED PROFESSOR
DEPARTMENT OF KINESIOLOGY AND HEALTH
DIRECTOR, CARDIAC NEUROSCIENCE LABORATORY
CENTER OF ALCOHOL STUDIES
RUTGERS STATE UNIVERSITY OF NEW JERSEY
PISCATAWAY, NJ 08854-8001

BRADLEY, KATHARINE ANTHONY, MD, MPH
SENIOR INVESTIGATOR
GROUP HEALTH RESEARCH INSTITUTE
SEATTLE, WA 98101

CARROLL, KATHLEEN M., PHD
ALBERT E. KENT PROFESSOR OF PSYCHIATRY
DIRECTOR OF PSYCHOSOCIAL RESEARCH
DIVISION OF ADDICTIONS
DEPARTMENT OF PSYCHIATRY
YALE UNIVERSITY SCHOOL OF MEDICINE
WEST HAVEN, CT 06511

CHOI, DOO-SUP, PHD
PROFESSOR OF PHARMACOLOGY AND PSYCHIATRY
DEPARTMENT OF MOLECULAR PHARMACOLOGY
AND EXPERIMENTAL THERAPEUTICS
MAYO CLINIC COLLEGE OF MEDICINE
ROCHESTER, MN 55905

KELLY, JOHN F., PHD
ELIZABETH R. SPALLIN ASSOCIATE PROFESSOR OF
PSYCHIATRY IN ADDICTION MEDICINE AT HARVARD
MEDICAL SCHOOL
FOUNDER AND DIRECTOR, RECOVERY RESEARCH
INSTITUTE
ASSOCIATE DIRECTOR, CENTER FOR ADDICTION MEDICINE
MASSACHUSETTS GENERAL HOSPITAL
BOSTON, MA 02114

MURPHY, JAMES G., PHD
PROFESSOR AND DIRECTOR OF CLINICAL TRAINING
DEPARTMENT OF PSYCHOLOGY
UNIVERSITY OF MEMPHIS
MEMPHIS, TN 38152

RAY, LARA A., PHD
PROFESSOR
DEPARTMENT OF PSYCHOLOGY
UNIVERSITY OF CALIFORNIA, LOS ANGELES
LOS ANGELES, CA 90095

ZWEBEN, ALLEN, PHD
ASSOCIATE DEAN, RESEARCH AND ACADEMIC AFFAIRS
PROFESSOR OF SOCIAL WORK
SCHOOL OF SOCIAL WORK
COLUMBIA UNIVERSITY
NEW YORK, NY 10027

SCIENTIFIC REVIEW OFFICER

SRINIVAS, RANGA V., PHD
CHIEF, EXTRAMURAL PROJECT REVIEW BRANCH
EXTRAMURAL PROJECT REVIEW BRANCH
NATIONAL INSTITUTE ON ALCOHOL ABUSE AND
ALCOHOLISM
NATIONAL INSTITUTES OF HEALTH
BETHESDA, MD 20892

EXTRAMURAL SUPPORT ASSISTANT

STRINGFIELD, DONNA
EXTRAMURAL SUPPORT ASSISTANT
OFFICE OF EXTRAMURAL ACTIVITIES
NATIONAL INSTITUTE ON ALCOHOL ABUSE AND
ALCOHOLISM
NATIONAL INSTITUTES OF HEALTH
ROCKVILLE, MD 20852

Consultants are required to absent themselves from the room during the review of any application if their presence would constitute or appear to constitute a conflict of interest.
